# Quantitative and qualitative shifts in defensive metabolites define chemical defense investment during leaf development in *Inga*, a genus of tropical trees

**DOI:** 10.1002/ece3.1896

**Published:** 2016-01-08

**Authors:** Natasha L. Wiggins, Dale L. Forrister, María‐José Endara, Phyllis D. Coley, Thomas A. Kursar

**Affiliations:** ^1^ Department of BiologyUniversity of UtahSalt Lake CityUtah; ^2^ School of Biological SciencesUniversity of TasmaniaHobartTas.Australia; ^3^ Smithsonian Tropical Research InstituteBalboaPanama

**Keywords:** Foliar chemistry, *Inga*, leaf development, metabolomics, plant defense traits, secondary metabolites

## Abstract

Selective pressures imposed by herbivores are often positively correlated with investments that plants make in defense. Research based on the framework of an evolutionary arms race has improved our understanding of why the amount and types of defenses differ between plant species. However, plant species are exposed to different selective pressures during the life of a leaf, such that expanding leaves suffer more damage from herbivores and pathogens than mature leaves. We hypothesize that this differential selective pressure may result in contrasting quantitative and qualitative defense investment in plants exposed to natural selective pressures in the field. To characterize shifts in chemical defenses, we chose six species of *Inga,* a speciose Neotropical tree genus. Focal species represent diverse chemical, morphological, and developmental defense traits and were collected from a single site in the Amazonian rainforest. Chemical defenses were measured gravimetrically and by characterizing the metabolome of expanding and mature leaves. Quantitative investment in phenolics plus saponins, the major classes of chemical defenses identified in *Inga*, was greater for expanding than mature leaves (46% and 24% of dry weight, respectively). This supports the theory that, because expanding leaves are under greater selective pressure from herbivores, they rely more upon chemical defense as an antiherbivore strategy than do mature leaves. Qualitatively, mature and expanding leaves were distinct and mature leaves contained more total and unique metabolites. Intraspecific variation was greater for mature leaves than expanding leaves, suggesting that leaf development is canalized. This study provides a snapshot of chemical defense investment in a speciose genus of tropical trees during the short, few‐week period of leaf development. Exploring the metabolome through quantitative and qualitative profiling enables a more comprehensive examination of foliar chemical defense investment.

## Introduction

Plants use a variety of strategies to reduce herbivory, which include morphological, phenological, developmental, and/or chemical defenses (Mithöfer and Boland 2012; Turley et al. 2013; Lamarre et al. 2014; Endara et al. 2015). That the selective pressures imposed by herbivores are correlated with plant defense traits is consistent with an evolutionary arms race in plant–herbivore systems (Becerra et al. 2009; Kursar et al. 2009; Endara et al. 2015). Research based on the framework of an arms race has improved our understanding about the patterns of defense investment and the selective pressures that have led to the variety of defensive strategies across species. However, defenses are not static, as plants are under different regimes of selective pressures during their lifetimes (Karban and Baldwin 1997; Agrawal and Karban 1999; Brenes‐Arguedas and Coley 2005; Boege and Marquis 2006; Brenes‐Arguedas et al. 2008; Barton and Koricheva 2010; Koricheva and Barton 2012; Karolewski et al. 2013; Abdala‐Roberts et al. 2014). Here, we explore how secondary metabolites change across even shorter timescales, specifically across weeks during the development of a single leaf.

From expansion to maturity, the antiherbivore defenses of leaves undergo considerable qualitative changes. During the first few weeks after bud break, young leaves are expanding, and this growth requires a high investment in proteins. Because cell walls do not lignify until the leaf has reached full size, expanding leaves lack toughness. Thus, young leaves are constrained to be tender and nutritious and therefore are preferred by herbivores. In tropical rainforests, more than 70% of the total herbivory that a leaf experiences throughout its lifespan occurs during the few weeks of leaf expansion (Coley and Aide 1991; Coley and Barone 1996). When the leaf reaches full size, it quickly lignifies, nutritional levels decline, and herbivory drops 25‐fold (Kursar and Coley 2003). During the typical two‐ to four‐year lifespan of a leaf in the understory, there is little change in leaf traits and herbivory remains low (Coley and Barone 1996; Kursar and Coley 2003). Because expanding leaves do not have the benefit of two of the most effective defenses, high toughness, and low nutrients, they must depend upon alternate defense strategies against herbivory. Besides secondary chemistry, expanding leaves also invest in other defensive mechanisms including delayed chloroplast development, rapid leaf expansion, population‐level synchrony in leaf flushing, and/or the production of extra‐floral nectar to attract predacious ants (Kursar and Coley 1992; Kursar et al. 2009; Marazzi et al. 2013). Hence, expanding leaves are under stronger selection by herbivores than mature leaves. In addition, because younger tissues have higher value to the plant, higher defenses, relative to more mature tissues, are predicted.

In this study, we focus on differences between expanding and mature leaves in the expression of secondary metabolites, a defense trait that plays a key role in plant–herbivore interactions. Each leaf must make the transition from bud to maturity, yet specific details of both the quantitative and qualitative changes in secondary metabolites, and thus the metabolome, during development are seldom thoroughly studied (although this approach has been recently embraced by researchers for applied applications; e.g., Shuib et al. 2011; Plischke et al. 2012; Müller et al. 2015). With the advent of metabolomic approaches (Patti et al. 2012), we can address these questions with a more detailed examination of plant secondary metabolites than has been previously available. Here, we apply ultra‐performance liquid chromatography coupled with high‐resolution detection of mass (LC‐MS), to the analysis of metabolites of intermediate polarity (principally flavonoids, tannins, and saponins). We employ untargeted metabolomics, whereby all metabolites, most of which are not yet identified, are detected, characterized by elution time and mass, and quantified. Untargeted metabolomics measures the relative abundance of thousands of ions. The combined elution time and mass‐to‐charge ratio of each ion is unique and is termed a “feature.” As each feature is unique, samples can be compared by sorting thousands of features present in each sample into those that are present in many samples versus those that are present in a limited set of samples. Thus, current LC‐MS technology provides us with a chemical fingerprint of foliar defense profiles, that is, both comprehensive and quantitative. Typically, previous studies have examined a small subset of chemically similar compounds (e.g., alkaloids) or used spectrophotometric assays to provide a single percent investment for an entire class (e.g., total phenolics). The richness of information about, for example, the suite of phenolic compounds, would thus be overlooked. Therefore, the use of metabolomics enhances our understanding of plant defensive chemistry with a degree of detail previously unavailable, and holds much potential for the study of how plants interact with herbivores.

*Inga* contains a diversity of secondary metabolites, including phenolics, saponins, and nonprotein amino acids. Here, we focus on phenolics and saponins, molecules with intermediate polarity, as they are the most diverse and dominant secondary metabolites in *Inga* (Kursar et al. 2009). Moreover, these metabolites had substantial detrimental effects in a laboratory bioassay with a lepidopteran herbivore (Coley et al. 2005; Lokvam and Kursar 2005; Lokvam et al. 2006), demonstrating that *in vivo* levels of these metabolites are highly toxic. To characterize shifts in chemical defenses and to assess the universality of patterns, we chose six species of *Inga,* an abundant and speciose genus of trees found throughout the New World tropics. The focal species represent diverse chemical, morphological, and developmental defense traits and were collected from a single site in the Amazonian rainforest.

We hypothesize that (1) foliar chemical defense investment should be greater during the expansion stage when leaves are more vulnerable to herbivores, than in leaves that have matured and toughened. We also expect that, due to selection by herbivores, (2) the suite of secondary metabolites expressed in expanding and mature leaves will differ. For example, just as novel or divergent defenses among species may promote escape from specialized herbivores (Kursar et al. 2009; Endara et al. 2015; Richards et al. 2015), divergence between the defenses of expanding and mature leaves of the same species may also be advantageous. Lastly, assuming that polymorphisms or intraspecific variation in defenses provides escape from herbivores and given high damage to expanding leaves, we predict (3) greater intraspecific variation in defenses for expanding than for mature leaves.

## Materials and Methods

### Field collections

**Figure 1:**
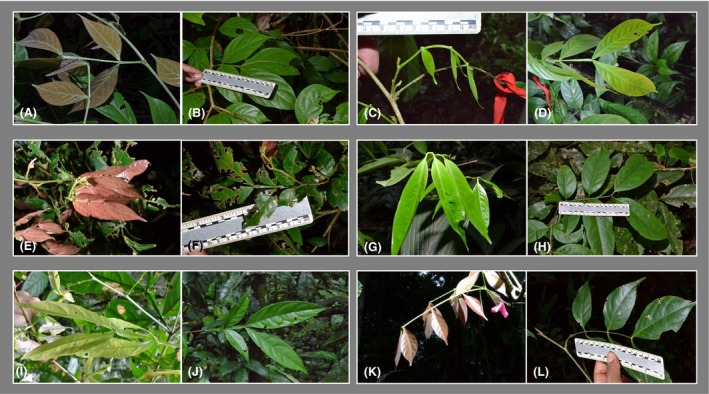
Images of expanding and mature leaves for *Inga marginata* (A,B), *I. acreana* (C,D), *I. auristellae* (E,F), *I. tenuistipula* (G,H), *I. umbellifera* (I,J), and *I. laurina* (K,L).

Expanding and mature leaves of *Inga* (Fig. 1) were collected in 2013 and 2014 at the Tiputini Biodiversity Station located in the eastern Ecuadorian Amazon in the Yasuní Biosphere Reserve (00°37′05″S, 76°10′15″W, c. 250 m above sea level). The 650‐ha lowland forest is terra firme, and the climate is tropical and humid, with a mean annual temperature of 24°C and rainfall of 3250 mm (Marsh 2004). The genus *Inga* is one of the most abundant and speciose genera at most lowland forests in the New World (Valencia et al. 1994; ter Steege et al. 2013).

We collected expanding and mature leaves from saplings that were approximately 0.5–4 m tall. For each of our six focal species, we collected five expanding and five mature leaves (*n *=* *10 samples per species) from 7 to 10 trees. This represents five replicates per leaf age class per species (Appendix S1). Fifty‐five of the 60 samples were from completely or partly shaded sites, and only three expanding‐ and two mature leaf samples were collected from sunny light gaps. However, close examination of leaf collection details (Appendix S1) and the dendrogram created from the hierarchical cluster analysis (Results; Appendix S9) shows no evidence for light levels influencing leaf sample clustering. This is consistent with previous studies on *Inga* showing no response of expanding leaves to shaded‐versus‐gap conditions (Bixenmann 2011; Shuib et al. 2011). Expanding leaves were collected when they were approximately 50% of full size (15–90% expansion range, Appendix S1). Because secondary metabolite investment changes during expansion (Brenes‐Arguedas et al. 2006), and because it is not always possible to obtain leaves at precisely 50% expansion, this could cause some intraspecific variation. Mature leaves were full size, completely tough and green, and free of epiphylls and, based on 35 years of studying leaf development, we estimate these to be 6–12 months of age. A total of 60 samples were collected from 45 trees. As highlighted in the map of collection sites (Appendix S2), some samples of expanding leaves were collected from the same individual tree as mature leaf samples. A total of 15 pairs of leaves were collected for expanding and mature leaves from the same tree (paired collections), and 15 expanding and mature leaf samples were collected from different trees of the same species (nonpaired collections).

In order to minimize spatial correlation between sampling units and any effects of clumping, sampling was conducted across 9 km^2^ of the reserve (Appendix S2). The average distance between plants of a given species was 1087 m (Appendix S3), much larger than the distances demonstrating spatial autocorrelation of tropical trees (Condit et al. 2000; Fuller and Enquist 2011). Spatial autocorrelation is strongest in tropical tree distributions at distances <20 m (Condit et al. 2000); thus, an average distance of 1000 m between sampled individuals should avoid, at least in part, this problem.

### Species selection

In order to obtain results that are likely to be broadly applicable, we selected species for which the expanding leaves have distinct chemical defenses (saponins, phenolics, and tyrosine), and developmental defenses (leaf expansion rate, chlorophyll content; Table 1). Two species per chemical defense class category were chosen (Appendix S4, 5).

**Table 1:** Chemical and developmental traits of *Inga* species

Species	Distinguishing chemical defense class	Leaf expansion strategy	Leaf expansion rate^a^ (% per day)	Chlorophyll content^a^^,^ b (mg/m^2^)
*Inga marginata* Willd.	Saponins, phenolics	Fast	58.2 ± 15.9 (37)	62 ± 12 (23)
*Inga acreana* Harms	Saponins, phenolics	Slow	23.7 ± 3.4 (40)	94 ± 17 (14)
*Inga auristellae* Harms	Phenolics, saponins	Fast	58.3 ± 26.1 (35)	41 ± 11 (17)
*Inga tenuistipula* Ducke	Phenolics	Slow	31.0 ± 8.7 (29)	164 ± 26 (8)
*Inga umbellifera* c (Vahl) Steud ex DC.	Tyrosine, phenolics	Fast	42.6 ± 3.9 (20)	49 ± 13 (13)
*Inga laurina* (Sw.) Willd.	Tyrosine gallates	Fast	57.2 ± 17.7 (38)	56 ± 16 (10)

a Values are means ± SD, with the sample sizes presented in parentheses.

b Chlorophyll content was determined as described in Appendix S4.

c For *I. umbellifera*, leaf expansion rate was estimated from the chlorophyll content using data on expansion and chlorophyll content (see Kursar and Coley 2003).

Species are ordered by their “distinguishing chemical defense class,” and this order will be followed throughout the manuscript.

### Chemical analyses

In the field, leaf samples were dried at room temperature using fans and silica gel for 24–48 h, transported to the University of Utah, and stored in a −20°C freezer. In Utah, samples were vacuum‐dried for 24 h to remove residual water and ground using a Retsch^®^ Ball Mill (MM 200, Haan, Germany) at 30 Hz for 20–30 sec. Samples <300 mg dry weight were ground in a 2‐mL cryovial with two nickel‐plated ball bearings (1/8 inch, VXB Ball Bearings) for 30 sec in a Wig‐L‐Bug (International Crystal Laboratories, Garfield, NJ) with speed set at 38. We combined 100 ± 2.5 mg of ground leaves with 1.0 mL of extraction buffer (44.3 mmol/L ammonium acetate (pH 4.8):acetonitrile, 60:40, v/v), mixed for 5 min, and centrifuged at 13,800 × g for 5 min. The supernatant was retained. The pellet was re‐suspended in extraction buffer, mixed, and centrifuged again. The two supernatants were combined, yielding 2 mL crude extract. The pellet or marc was vacuum‐dried and weighed. For chromatographic analyses, the crude extract was diluted fivefold by combining 790 *μ*L of acetonitrile:water (60:40, v/v), 200 *μ*L crude extract, and 10 *μ*L internal standard (1 mg/mL biochanin A in acetonitrile:water, 50:50). Mature leaf samples were also analyzed as undiluted crude extract due to lower concentrations of metabolites.

Soluble metabolites were analyzed by ultra‐performance liquid chromatography and mass spectrometry (UPLC‐MS) using an Acquity UPLC^®^ *I‐Class* system and a Xevo^®^ G2 Q‐ToF MS equipped with LockSpray™ and an electrospray ionization source (Waters, Milford, MA). For LC conditions, 1 *μ*L of sample was analyzed using an Acquity^®^ BEH C18 1.7 *μ*m column (2.1 × 150 mm) fitted with a VanGuard Precolumn (2.1 × 5 mm; Waters). The mobile phases were water with 0.1% formic acid (Solvent A) and acetonitrile with 0.1% formic acid (Solvent B; Optima^®^ LC/MS grade, Fisher Scientific, Waltham, MA) with a flow rate of 0.5 mL/min and the column at 40°C. Gradient conditions are presented in Appendix S6. The MS conditions were as follows: capillary voltage 2.50 kV, sampling cone voltage 45 V, extraction cone voltage 3.5 V, source temperature 100°C, desolvation gas temperature 400°C, desolvation gas flow 600 L/h, negative ionization and resolution modes, *m/z* (mass to charge) ratio of 50–2000 Da, centroid mode, and a collision energy of 6 eV. In MS mode, the collision energy is set by the manufacturer (not user‐controlled) and functions to enhance sensitivity and resolution while avoiding fragmentation.

Metabolites that are covalently bound to the marc, here termed insoluble metabolites, were extracted from the marc using a butanol–HCl hydrolysis and then assayed for anthocyanidins. The marc was extracted with 1.5 mL of butanol–HCl and 0.05 mL Iron Reagent at 100°C for 50 min (Porter et al. 1985; Hagerman 2002). The digest was centrifuged for 5 min at 13,800 × g, the supernatant was diluted 5× with butanol–HCl, and the absorbance was measured at 550 nm. This was converted to mass using crude quebracho tannin as a standard (Harshaw Chemicals, Glasgow, see Coley 1983). The marc was re‐suspended in methanol and centrifuged, then repeated with water. The supernatants were discarded, and the marc was vacuum‐dried and weighed.

### Conversion and pre‐processing of LC‐MS data

The UPLC‐MS data were converted to tables with the retention time and intensity or total ion current (TIC) for each feature. Inspection of the chromatographic runs (Appendix S7) showed that the 1:5 dilutions of expanding leaf extracts and the undiluted mature leaf extracts were more readily comparable. Hence, statistical analyses were performed on these datasets (with the exception of the TIC data, Table 2). The UPLC‐MS raw data files were converted to Common Data Format (CDF, NASA/Goddard Space Physics Data Facility) using MassLynx (Waters). In order to identify then align features present in multiple samples, CDF files were processed using XCMS (Bioconductor v3.1 (Smith et al. 2006; Tautenhahn et al. 2008; Benton et al. 2010) in R v3.1.3 (R Development Core Team, 2015; Appendix S8). The following parameters were used: feature detection method “centWave” (ppm 10, snthresh 4); peak grouping method “density” (bw = 10); retention time correction method “loess”; peak integration method “chrom.” Preprocessing with XCMS was performed separately for each species, with five expanding and five mature leaf samples included as replicates in each set. The first 1.5 min of each run was removed as these data represent unretained, highly polar compounds. A value of 1 was added to all TIC values to remove zeros from the dataset.

**Table 2:** Total ion current for six species of *Inga*

Species	Total ion current^a^		
	Expanding leaves 1:5 dilution	Mature leaves 1:5 dilution	Mature leaves no dilution
*Inga marginata*	472 ± 49	151 ± 6***	1050 ± 60
*Inga acreana*	490 ± 2	568 ± 181***	774 ± 4
*Inga auristellae*	948 ± 84	291 ± 33***	1477 ± 448
*Inga tenuistipula*	418 ± 35	201 ± 69***	1077 ± 74
*Inga umbellifera*	169 ± 46	182 ± 118***	441 ± 152
*Inga laurina*	953 ± 151	145 ± 8***	1086 ± 205

A value of *P *<* *0.001 for the difference between expanding and mature leaves (Kruskal–Wallis test) is indicated by ***.

a Values are the mean total ion current divided by 10^4^ ± SD.

### Data normalization and scaling

Data filtering was performed using the interquartile range, selected as a robust estimate to filter data for untargeted metabolomics. Data were normalized using sample normalization by sum, and data scaling was performed using auto‐scaling.

### Statistical analyses

The following analyses were performed in the statistical programming language R v3.1.3 (R Development Core Team, 2015; Appendix S8) and guided by recent reviews on metabolomic data analysis (Vinaixa et al. 2012; Ren et al. 2015). The effect of leaf age on chemical similarity (assessing an average across species of 1646 ± 499 features, range 1133–2319) was analyzed using two approaches in which samples were not categorized as to developmental stage. As membership in each group was not defined *a priori*, these methods provide unbiased visualizations of whether or not samples are clustered based on the metabolome. A principal component analysis (PCA) was run for each species using the library muma (Gaude et al. 2013), followed by a hierarchical cluster analysis using the library pvclust (Borcard et al. 2012; Suzuki and Shimodaira 2014). Subsequently, in order to calculate a statistical measure of confidence for the selected clusters (Approximately Unbiased confidence levels), hierarchical cluster analysis was performed with 10,000 bootstrap iterations and multiscale resampling. For *I. marginata*,* I. auristellae*,* I. tenuistipula,* and *I. laurina*, we used the Ward clustering algorithm, and the median clustering method for *I. acreana* and *I. umbellifera*. Model selection was based on the relationship between the original distance matrix and the binary matrix representing the partitions in the cluster diagram. For this we used a cophenetic correlation. The clustering algorithm (i.e., single, ward, complete, centroid, median) with the highest cophenetic correlation was selected as the best one for each species. A correlation‐based dissimilarity matrix was used as a measure of the actual distance among samples.

In order to evaluate the validity of group assignments, we also performed a partial least squares‐discriminant analysis (PLS‐DA) in MetaboAnalyst 3.0 (Xia et al. 2015). All leaf samples were assigned to a group (species x leaf age) *a priori*. This approach provides the greatest separation among the groups. The *P*‐value generated from a permutation test assessed the significance of group separation. The *R* ^2^ value assessed the goodness‐of‐fit of the model to the data. A second statistic, *Q* ^*2*^, assesses agreement between the modeled and actual data after removal of some features and should be much greater than 0.5. Although PLS‐DA has a tendency to over‐fit models to data, this is not the case when *R* ^2^ does not greatly exceed *Q* ^*2*^ (Worley and Powers 2013).

We evaluated the possibility that the observed chemical differences within a species were due to differences among trees rather than due to leaf age. Across all species, a total of 15 pairs of leaves were collected for expanding and mature leaves from the same tree (paired collections), and 15 expanding and 15 mature leaf samples were collected from different trees of the same species (nonpaired collections; see Appendix S1 for a map of the collection sites). We divided the 60 samples into four groups based on paired versus nonpaired collections and leaf age, and compared these using PLS‐DA.

Area‐proportional Venn diagrams (Pacific Northwest National Laboratory, 2015) were used to represent the overlap in the number of secondary metabolites between expanding and mature leaves.

Differences in the masses of metabolites extracted from expanding and mature leaves, and in the remaining marc weight, were estimated using Welch's *t*‐tests for within‐species comparisons, and an ANOVA for a global species comparison. Univariate statistics were also performed on TIC (total ion current) comparisons, feature consistency, and the coefficient of variation using a Kruskal–Wallis test between expanding and mature leaves for each species. We correlated the TIC values for compounds that are shared between expanding and mature leaves, and present the associated *R* ^2^ values. The cutoff used for disregarding peaks that were not consistently detected in individual samples was set at TIC < 1000.

## Results

### Chemical defense profiles differ between leaf age classes

Focal species were chosen based, in part, on their divergent chemistry. In fact, a PLS‐DA of species (six groups and all 60 samples) separated all of the species with no confidence interval overlap (*R* ^*2*^ **= 0.96, *Q* ^*2*^ **= 0.92, *P *<* *0.01, Appendix S5), indicating distinct chemistries. Even species with similar classes of “distinguishing chemical defense” (saponins, phenolics or tyrosine in Table 1) were statistically distinct.

**Figure 2:**
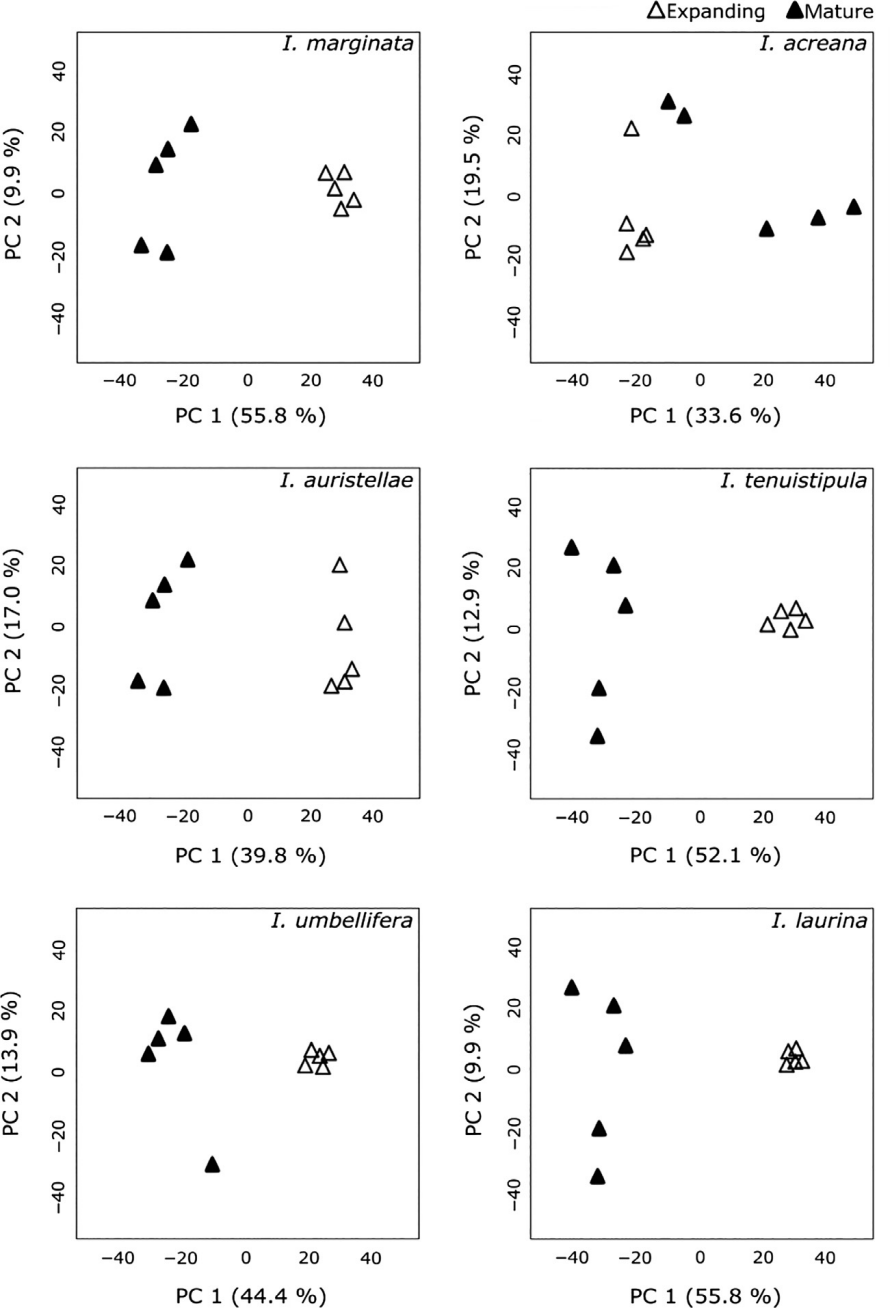
Principal component analysis plots for each species, with expanding and mature leaves denoted by “e” and “m,” respectively. Values are derived from the total ion current of the features obtained from UPLC‐MS analysis. Species are ordered by their “distinguishing chemical defense class” (saponins, phenolics, or tyrosine; see Table 1).

We analyzed the effect of leaf age on chemistry using PCA, clustering, and discriminant analysis. A PCA, in which age categories were not assigned, was run for each of the six species to visualize the similarity or divergence of the metabolomic profiles. For each species, the samples generally clustered into two distinct groups, one for expanding and one for mature leaves (Fig. 2). The top two components explained 53–66% of the variation. Hierarchical clustering analysis with bootstrap re‐sampling was used to estimate the confidence levels (probability values) for clusters. Strong bootstrapping support generally confirmed the patterns observed with PCA (Appendix S9).

**Figure 3:**
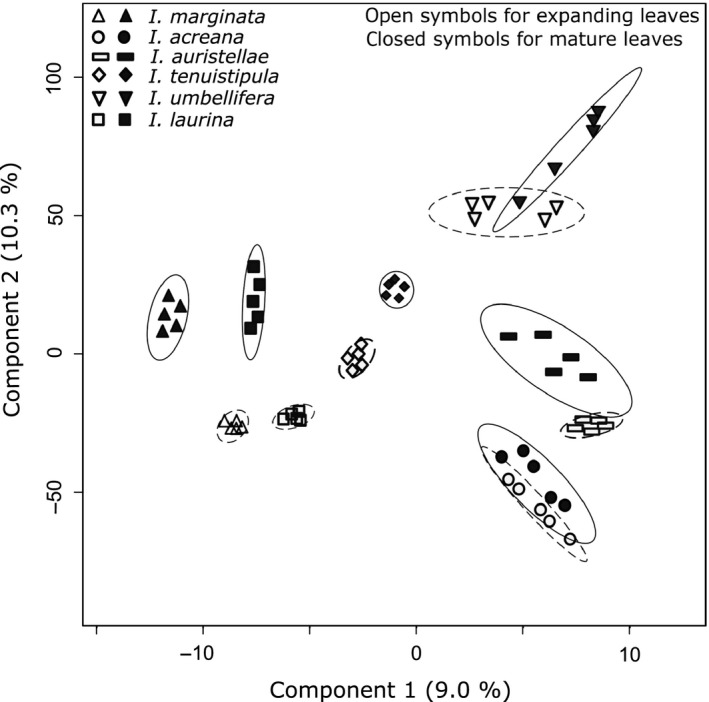
A two‐dimensional scores plot of a partial least squares‐discriminant analysis of the metabolite profiles of expanding and mature leaves from six species of *Inga* (*n *=* *5 samples per leaf age class per species). Age class is represented by open (expanding) or closed (mature) symbols, and 95% confidence intervals are indicated by a dashed (expanding) or solid (mature) line around each sample set. Values are derived from the total ion current of individual features obtained from UPLC‐MS analysis.

Lastly, we used discriminant analysis, in which samples are pre‐assigned to expanding or mature age classes for each species, to test the hypothesis that metabolite profiles distinguish species x leaf age. A PLS‐DA for species x leaf age (12 groups and all 60 samples) was significant (*R* ^2^ = 0.92, *Q* ^*2*^ **= 0.90, *P *<* *0.01, Fig. 3).

**Figure 4:**
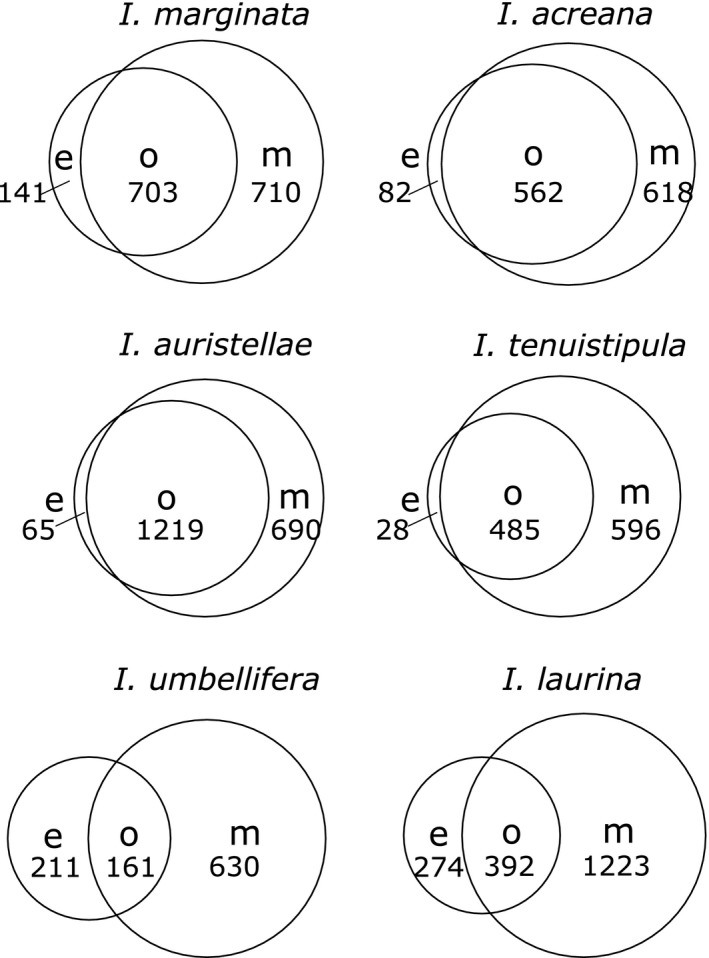
Venn diagrams showing the degree of overlap of chemical defenses between expanding and mature leaves. Values are derived from binary data to represent the presence/absence of unique features in expanding and mature leaves. Features that are shared between expanding and mature leaves are represented in the overlap between the two circles (where e‐expanding; m‐mature; o‐overlap).

When the effect of abundance was ignored, and instead, metabolites were scored as present/absent, we found that, for each species, the two leaf age classes shared some metabolites, but others were unique to either expanding or mature leaves. When averaged across species, expanding leaves contained 134 (±38 SE) unique features and mature leaves contained 758 (±95 SE) unique features (Fig. 4). When the total numbers of metabolites were considered (including those that were shared between leaf age class), expanding leaves contained a total of 721 (± 129 SE) features and mature leaves contained a total of 1344 (± 161 SE) features. A total of 587 (± 358 SE), or 22% of these features, were shared between both leaf age classes. There did appear to be species similarities in the proportion of metabolites present in each category (expanding, mature or overlap) for each “distinguishing chemical defense class” group (Table 1), as indicated by the observed patterns in the area‐proportional Venn diagrams (Fig. 4).

Expanding and mature leaves of each species showed more chemical differences than similarities. *I. marginata*,* I. laurina*, and *I. tenuistipula* have notable differences between the two age classes (Figs. 2, 3, Appendix S9). While *I. auristellae* and *I. umbellifera* showed clear separation in the PCA plots (Fig. 2), the more rigorous discriminant analysis and hierarchical clustering indicate minor overlap of some expanding and mature leaf samples (Fig. 3, Appendix S9). In contrast, analyses of the metabolomics data for *I. acreana* show that expanding and mature leaves are chemically similar (Figs. 2, 3, Appendix S9). Moreover, inspection of the chromatograms is consistent with this result (Appendix S7).

### Differences between expanding and mature leaves were greater than the differences among individual plants

We evaluated the possibility that the observed chemical differences within a species were due to differences among the individual trees from which samples were collected rather than due to leaf age (see Statistical Analysis for an explanation of paired versus nonpaired collections). Comparing the same leaf age, we found considerable overlap for paired versus nonpaired collections (Appendix S10). This indicates that the expanding and mature pairs from the same tree were not more chemically similar to each other than the collections from different trees. Therefore, the significant effects observed in the PLS‐DA appear to be driven more by the chemical profiles of expanding versus mature leaves than by differences among individual plants (*R* ^2^ = 0.52, *Q* ^*2*^ **= 0.40, *P *=* *0.03; Appendix S9).

### Quantitative investment in chemical defenses is higher in expanding leaves

**Figure 5:**
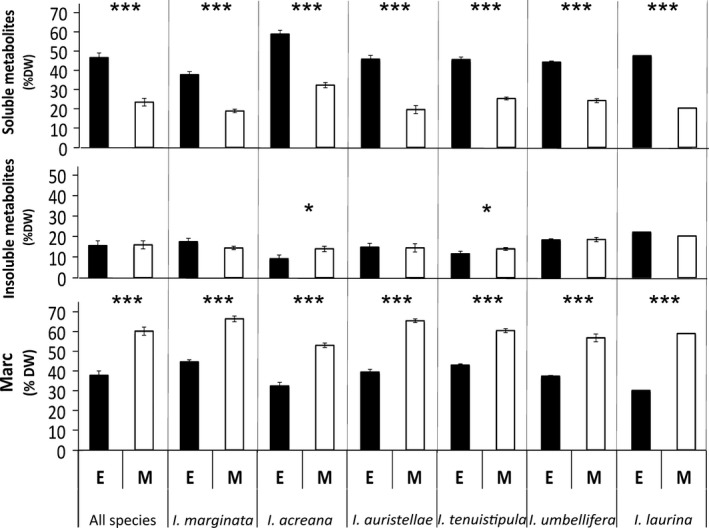
Soluble metabolites extracted using water:acetonitrile, insoluble metabolites extracted using butanol:HCl, and the remaining marc. Data are presented for expanding (E) and mature (M) leaves, where * denotes the level of significance (*P < 0.01; ***P < 0.001; Welch's *t*‐tests for within‐species comparisons; ANOVA for the “All Species” comparison).

Quantitative investment in phenolics and saponins was measured both gravimetrically and as relative concentration using total ion current (TIC). Gravimetrically, the mass of soluble metabolites was 46.0% of dry weight in expanding leaves versus 23.7% in mature leaves (*P *<* *0.01, Fig. 5). The ratio of the mass of soluble metabolites in expanding versus mature leaves was highest at 2.3 in *I. auristellae* and *I. laurina*, and lowest at 1.8 in *I. acreana*,* I. tenuistipula,* and *I. umbellifera*.

We also used the acid butanol method that was developed for the hydrolysis of proanthocyanidins in order to extract insoluble metabolites (metabolites covalently bound to components such as cell walls). These were assayed gravimetrically and spectrophotometrically. The mass of covalently bound metabolites did not differ substantially between leaf age classes, averaging 16.2% and 16.3% of dry weight for expanding and mature leaves, respectively (*P *=* *0.49; Fig. 5). The mass of the remaining insoluble components (the marc), primarily cell wall material, was greater in mature (60.3% ± 2.3 SE) than expanding leaves (37.9% ± 2.4 SE, *P *<* *0.01, Fig. 5). The spectrophotometric assay results were lower than expected and showed a different species‐specific pattern than the gravimetric results (Fig. 5, Appendix S10, see Discussion).

To quantify investment as relative concentration, we summed the TIC of all features present in a single chromatography run. Using TIC showed that the relative concentration of soluble chemical defense compounds in expanding leaves was approximately 2.5 times higher than in mature leaves (*P *<* *0.001, Table 2). By species, the ratio ranged from 0.9 for *I. acreana* to 6.6 for *I. laurina*.

We also examined the TIC data for the strength of correlations between the concentrations of compounds that were shared in expanding and mature leaves (using the 1:5 dilution data for all comparisons). Results generally indicate no strong correlation in the concentrations of shared compounds (*R* ^2^ range 0.01–0.28), with the exception of the two “saponin”‐containing species (Table 1) *I. marginata* and *I. acreana* (*R* ^2^ = 0.61 and 0.65, respectively, Table 3).

**Table 3:** Quantitative and qualitative measures of feature correlation and variability between and within *Inga* leaf age classes

Species	Leaf age class	TIC feature correlation^a^ ([exp]:[mat])	Feature consistency^b^ (%)	Coefficient of variation^c^ (%)
*Inga marginata*	Expanding	*R* ^2^ = 0.61	56.9***	46.9***
	Mature		50.2	54.9
*Inga acreana*	Expanding	*R* ^2^ = 0.65	8.9***	98.5***
	Mature		1.1	120.3
*Inga auristellae*	Expanding	*R* ^2^ = 0.01	60.8***	38.3***
	Mature		43.2	55.7
*Inga tenuistipula*	Expanding	*R* ^2^ = 0.28	63.2***	43.6***
	Mature		54.8	59.3
*Inga umbellifera*	Expanding	*R* ^2^ = 0.01	33.2***	70.2***
	Mature		17.4	92.6
*Inga laurina*	Expanding	*R* ^2^ = 0.01	54.2***	55.2***
	Mature		31.7	77.4

a TIC feature correlation relates feature abundance between the two age classes for the six species of *Inga*. The total ion current of every feature was averaged over all five samples, and the averages for expanding leaves were plotted versus mature leaves. A higher *R* ^2^ indicates more similarity between expanding and mature leaves.

b The “Feature consistency” parameter is a qualitative measure of variability. All features with TIC >10^3^ were counted as present and those <10^3^ as absent. This parameter is the percent of features for which that feature was present in all five samples, where a lower value indicates more variability.

c “Coefficient of variation” is a quantitative measure of variability. Within each age class, CV was calculated for the TIC of each feature then averaged – higher values indicate greater variability.

A *** indicates leaf age classes differed (*P *<* *0.001; Kruskal–Wallis test).

### Developmental shifts in chemistry do not correlate with chemical class or leaf expansion strategy

The differences between expanding and mature leaf chemistries did not correlate with any traits in Table 1. For qualitative differences in chemistry, the three species with expanding leaves that show notable differences are from all three chemical classes examined, and both leaf expansion strategies (Table 1). The two species with minor expanding/mature overlap in chemistry are from different chemical classes, but both have high rates of leaf expansion. *I. acreana*, with major overlap, has slow leaf expansion. For quantitative differences in chemistry, species were not highly differentiated and no conclusions can be drawn. Our results suggest that neither chemical class or expansion strategy correlates with developmental shifts in secondary metabolites.

### Intraspecific variation in chemical defenses is lower in expanding leaves

To investigate polymorphism and plasticity within each species, we evaluated the degree of feature consistency as a qualitative measure of variability. Using presence/absence data, the percent of features that were present in all five samples was 46% for expanding versus 33% for mature leaves when averaged by species (Table 3). Hence, mature leaves of the same species showed greater qualitative variability.

As a quantitative assessment of intraspecific variation, we calculated the coefficient of variation (CV) of the TIC of each feature, then averaged this CV for each species x age sample (Table 3). Averaging across the six species, the CVs are 58% for expanding and 77% for mature leaves. Hence, mature leaves showed more variation in ion counts (*P *<* *0.01, Table 3). Thus, qualitatively and quantitatively, expanding leaves were more similar than mature leaves of the same species.

## Discussion

Investment in chemical defense is an important strategy for plants combating herbivory (Fraenkel 1959; Ehrlich and Raven 1964; Becerra et al. 2009; Kursar et al. 2009; Endara et al. 2015; Richards et al. 2015). Theory predicts that young leaves, which require high levels of nutrients, have low lignification during growth and have higher value to the plant, invest more in chemical defenses against herbivory than mature leaves (Kursar and Coley 2003). Recent advances in metabolomics enable us to accurately measure the quantitative and qualitative defensive profiles of leaves. Here, we investigate the complexities of chemical defense investment at a timescale relevant to leaf life history, from expansion to maturity.

### Investment in secondary metabolites is exceedingly high and changes during leaf development

Multiple factors can affect the leaf metabolome including developmental, ontogenetic, spatiotemporal, and environmental factors, and it is often difficult to tease these apart (Lowman and Box 1983; Puttick 1986; Williams et al. 1997; Kause et al. 1999; Jeong et al. 2004; Close et al. 2005; Watanabe et al. 2013; Kjaer et al. 2014; Rivas‐Ubach et al. 2014). However, the switch to toughness as the major defense of mature leaves may be the main factor selecting for higher defense investments in young leaves (Coley and Barone 1996). Our results support this, as soluble chemical defenses were 1.9‐fold higher for expanding versus mature leaves and constituted a remarkable 46% of the total dry weight. Such changes in defenses could be related to the fact that expanding leaves are highly vulnerable to herbivores (Coley and Barone 1996). Other studies on woody plants also generally support higher concentrations of secondary metabolites for young leaves (Coley 1983; Mihaliak and Lincoln 1989; Gleadow and Woodrow 2000; Brenes‐Arguedas et al. 2006; Loney et al. 2006; Webber and Woodrow 2008; Koricheva and Barton 2012). However, a meta‐analysis (Koricheva and Barton 2012) found that some chemical classes show no change (cyanogenic glycosides and condensed tannins) or were higher in mature than in young leaves (sesquiterpenes).

There was also considerable investment in insoluble metabolites, with both expanding and mature leaves averaging 16% of dry weight (DW). Adding insoluble metabolites to the soluble mass gives a total, average mass of 62% metabolites in expanding leaves. Previous research on expanding leaves of two species of *Inga* from Panama, using more extensive extractions with organic solvents, obtained similar results. In that study, the fraction of intermediate polarity, phenolics and saponins, yielded about 30% of leaf dry weight for both species and the sum of all fractions was 48% DW and 61% DW for the two species (Lokvam and Kursar 2005). These values compare well with our results using a single extraction (one solvent) that yielded a mass averaging 46% DW (Fig. 5). The masses of insoluble metabolites extracted with butanol–HCl in the previous study, 17% and 21% of dry weight, were very similar to the value reported here of 16% DW on average. These results demonstrate that independent experiments yield similar, very high, masses of secondary metabolites in expanding leaves.

Although metabolites per dry weight may be the most relevant normalization of antiherbivore defense, these also can be quantified on the basis of leaf area or leaf volume. Given that dry weight approximately doubles and soluble metabolites per dry weight halve (Fig. 5) after the end of leaf expansion, soluble metabolites per area or volume would actually be similar for expanding and mature leaves. For insoluble metabolites, mature leaves would have about twofold more mass per area or volume. These estimates suggest that metabolites are synthesized at a steady rate as leaves expand and that some biosynthesis occurs after the end of leaf expansion. A study of soluble metabolites reported this pattern for *Inga goldmanii* whereas *I. umbellifera* showed much less investment late in leaf expansion and in maturity (Brenes‐Arguedas et al. 2006).

Our findings that insoluble metabolites make up between 9% and 23% of dry weight are supported by previous studies (Giner‐Chavez et al. 1997; Kraus et al. 2004; Lokvam and Kursar 2005). However, the chemical nature of these compounds is not clear. The butanol/HCl‐spectrophotometric assay for condensed tannins and the gravimetric results differed considerably. For example, *I. laurina* gave the lowest insoluble metabolites in the spectrophotometric assay and was the highest in the gravimetric analysis. In contrast, the highest in the spectrophotometric assay were *I. acreana* and *I. tenuistipula*, which were among the lowest in the gravimetric analysis. Such large differences in the spectrophotometric and gravimetric results suggest that species differ considerably in the chemical nature of the metabolites that are extracted from the marc using butanol–HCl. Clearly, the insoluble metabolites need to be better characterized in terms of composition and function.

In *Inga*, there is evidence that insoluble metabolites are toxic to insect herbivores and thus may function as a defense. We carried out feeding trials using caterpillars of *Heliothis virescens*, a moth having a tropical distribution and a generalized diet. We showed that when the marc along with any insoluble metabolites was incorporated as a realistic fraction, 35% of total dry weight of the diet, the marc was highly toxic (Appendix S12). For two plant species, we also found that marc with the insoluble metabolites removed was considerably less toxic than the marc plus insoluble metabolites. These experiments demonstrate that at least some of the mass extracted from the marc with butanol–HCl includes toxic metabolites. Although potentially toxic, our data show no differences in insoluble metabolite content between leaf age classes. This may be driven, in part, by physiological limitations to the amount of metabolites that can be sequestered in insoluble forms, in addition to posited alternative roles played by insoluble metabolites (McKey 1979; Moore and Jung 2001; Donaldson et al. 2006). Given their toxicity, sequestration in insoluble forms may permit high levels of accumulation while avoiding autotoxicity.

The exceedingly high investment in secondary metabolites by *Inga* reflects how the chemical arms race between plants and herbivores has influenced the defenses of tissues that are both vulnerable and of high value, particularly expanding leaves. We speculate that selection by herbivores may have pushed the accumulation of secondary compounds to the limit. Given that leaves must avoid autotoxicity and are involved in many essential functions including growth and photosynthesis, it may not be physiologically possible to invest any more in defenses without compromising other aspects of metabolism. Consistent with this idea is that there was negligible induction of phenolics and saponins in herbivore‐damaged compared to undamaged treatments of expanding *Inga* leaves (Bixenmann 2011).

### Profiles of defensive chemicals differ between leaf age classes

Recent advances in technology and bioinformatics provide us with previously unexplored profiles of the leaf metabolome, advancing our understanding of defensive metabolites beyond simply examining total concentrations of an entire class of compounds. Here, we have used unique features to characterize the suite of qualitative differences in defensive metabolites of wild plants.

The chemical profiles of expanding and mature leaves are generally quite different for each of the focal species, with the exception of *I. acreana*. Furthermore, although mature leaves had lower total investments, we found that they contained 72% more unique features, and more secondary metabolites overall than did the expanding leaves (Fig. 4). If metabolites in expanding leaves are not readily or completely catabolized, then metabolite diversity would increase with leaf age.

Of the possible explanations for the large observed shift that occurs in the short timeframe of only a few weeks, we discuss three. First, we posit that after leaf expansion, when the leaf is effectively defended by highly lignified cell walls (toughness), some defensive metabolites may be catabolized and used for a different purpose. This idea is supported by a previous finding that a toxic primary metabolite, tyrosine, which is highly expressed in expanding leaves, decreases considerably in mature leaves (Lokvam et al. 2006). While the role of tyrosine in proteins and its transformations in primary metabolism are well known, the extent to which secondary metabolites can be catabolized remains unclear.

Another explanation for the age differences in metabolites could be the cost of or the avoidance of autotoxicity, in which different compounds may be favored at different stages of leaf development. Autotoxicity and sequestration of plant secondary metabolites present potential physiological costs. These costs may be avoided if a nontoxic constitutive defense can be used (e.g., toughness with leaf maturity). Alternatively, the cost may be reduced if lower herbivore pressure or more unpredictable herbivory leads to the evolution of lower constitutive defenses (Agrawal and Karban 1999). In addition, the cost of sequestering metabolites over several weeks may be much less than that summed over the leaf's lifetime (years). This factor would select for a transition to mature‐leaf metabolites that have a low cost of sequestration. Interestingly, expanding leaves could, in theory, have compounds with a broader range of costs associated with sequestration and autotoxicity because of the short time frame. This would predict relatively less chemical diversity for mature leaves, the opposite of what is observed.

A third explanation for the observed shift in chemical profiles may be because herbivores attacking expanding and mature leaves are distinct, potentially requiring distinct secondary metabolites; that is, herbivores that can attack tough, low‐protein, mature leaves may not have the adaptations necessary for successful development on expanding leaves of the same species. In contrast, herbivores that can develop on expanding leaves must be adapted to the developmental defenses of plants, such as the capacity to complete larval development during the short window of time before leaf toughening (Aide and Londoño 1989). Moreover, the female adult is confronted with the challenges of finding and ovipositing on ephemeral buds. The fact that the chemistries of expanding and mature leaves are different probably represents another major constraint, such that herbivores must specialize on either expanding or mature leaves of the same species.

### Intraspecific variation in chemical defenses is greater in mature leaves

We predicted that intraspecific variation in secondary metabolites would be greater for expanding than mature leaves. This is an extension of the observation that, among coexisting species, novel or divergent defenses are found more frequently than expected by chance, possibly because this provides partial escape from herbivores (Becerra et al. 2009; Kursar et al. 2009). Additionally, sister species are not more similar in defenses, suggesting strong selection for divergent secondary metabolites (Kursar et al. 2009; Sedio 2013; Endara et al. 2015). Because herbivore damage is so high on the expanding leaves, we predicted an advantage of divergent defenses. Although the same argument could apply to mature leaves, the advantages of divergent metabolites might be less because they have high toughness, low nutrients, and low herbivory.

However, in this study, both qualitative and quantitative metrics of metabolite variation were greater for mature leaves (as demonstrated by feature consistency and the coefficient of variation, Table 3). This assessment of intraspecific variation is consistent with the patterns observed in the cluster analyses, with expanding leaves demonstrating tighter clusters than mature leaves (Figs. 2, 3). Intraspecific variation among mature leaves may reflect plastic responses over a longer time frame, months to years, to environmental conditions (particularly light, see Appendix S1). Although we expected selection by herbivores to favor divergent defenses in expanding leaves, the observed low levels of intraspecific variation suggest that leaf development is canalized or physiologically buffered such that a consistent phenotype is produced despite variation in the environment or in genotype. Other studies have shown similar canalization for expanding leaves (Bixenmann 2011; Sinimbu et al. 2012).

## Conclusions

Recent advances in metabolomics and bioinformatics, and their intersection with ecological and evolutionary questions, provide us with a unique platform upon which to investigate shifts in the chemical defenses of developing leaves. For *Inga*, we found that the chemical profiles as well as total investment in secondary metabolites differed between leaf developmental stages for all six species, regardless of whether they made primarily saponins, phenolics, or tyrosine‐based defensive metabolites. Expanding leaves demonstrated a higher quantitative investment in chemical defenses, while mature leaves contained a greater number of defensive metabolites and a greater degree of variability between samples. The observed shifts in quantitative chemical defense investment between leaf developmental stages support the theory that expanding leaves rely more upon chemical defense as an antiherbivore strategy than do mature leaves. Lower qualitative variation in chemical defense metabolites in young leaves provides support for the theory that leaf development is canalized.

## Conflict of Interest

None declared.

## Data Accessibility

All data are included in the manuscript and supporting information.

## Supporting information


**Appendix S1.** Details of leaf sample collections from the field (collection sites are presented in Appendix S2).
**Appendix S2.** Map of collection sites at the Tiputini Biodiversity Station in Ecuador.
**Appendix S3.** Distances between trees of *Inga* species that were sampled for expanding and mature leaves.
**Appendix S4.** Chlorophyll content in expanding leaves.
**Appendix S5.** A two‐dimensional scores plot of a partial least squares‐discriminant analysis of the metabolite profiles of all six species of *Inga*.
**Appendix S6.** Elution gradient used for UPLC‐MS runs.
**Appendix S7.** Chromatograms for expanding and mature leaves for the six *Inga* species.
**Appendix S8.** R code for statistical analyses of the LC‐MS data.
**Appendix S9.** Hierarchical cluster dendrograms of the metabolites of expanding and mature leaves from six species of *Inga*.
**Appendix S10.** Two‐dimensional score scatter plot from a partial least squares‐discriminant analysis fitted to the metabolite profiles of leaves sampled as a “paired sample collection” versus a “nonpaired sample collection.”
**Appendix S11.** Insoluble metabolites assayed as condensed tannin equivalents.
**Appendix S12.** Toxicity of the marc for species of *Inga* from Barro Colorado Island, Panama.
