# Supplementary material for: Quantitative and qualitative shifts in defensive metabolites define chemical defense investment during leaf development in Inga, a genus of tropical trees
Source: Ecol Evol. 2016 Jan 8;6(2):478–92. doi: 10.1002/ece3.1896 (PMC4729263; doi:10.1002/ece3.1896)
Supplement: Supplementary file 1 — Appendix S1. Details of leaf sample collections from the field (collection sites are presented in Appendix S2). Appendix S2. Map of collection sites at the Tiputini Biodiversity Station in Ecuador. Appendix S3. Distances between trees of Inga species that were sampled for expanding and mature leaves. Appendix S4. Chlorophyll content in expanding leaves. Appendix S5. A two‐dimensional scores plot of a partial least squares‐discriminant analysis of the metabolite profiles of all six species of Inga. Appendix S6. Elution gradient used for UPLC‐MS runs. Appendix S7. Chromatograms for expanding and mature leaves for the six Inga species. Appendix S8. R code for statistical analyses of the LC‐MS data. Appendix S9. Hierarchical cluster dendrograms of the metabolites of expanding and mature leaves from six species of Inga. Appendix S10. Two‐dimensional score scatter plot from a partial least squares‐discriminant analysis fitted to the metabolite profiles of leaves sampled as a “paired sample collection” versus a “nonpaired sample collection.” Appendix S11. Insoluble metabolites assayed as condensed tannin equivalents. Appendix S12. Toxicity of the marc for species of Inga from Barro Colorado Island, Panama. [file ECE3-6-478-s001.docx]

## Supplementary material

The following Supplementary material is available for this article:

**Appendix 1** Details of leaf sample collections from the field (collection sites are presented in Appendix 2).

**Appendix 2** Map of collection sites at the Tiputini Biodiversity Station in Ecuador. The bold line is the Rio Tiputini and the gray lines are trails. Inset: approximate location of Tiputini in Ecuador.

**Appendix 3** Distances between trees of *Inga* species that were sampled for expanding and mature leaves.

**Appendix 4** Chlorophyll content in expanding leaves (values are presented in Table 1).

**Appendix 5** A two-dimensional scores scatter plot of a partial least squares-discriminant analysis of the metabolite profiles of all six species of *Inga* (*n*=10 samples per species). Species are represented by shapes, and 95 % confidence intervals are indicated by a solid line around each sample set. Values are derived from the total ion current of individual features.

**Appendix 6** Elution gradient used for UPLC-MS runs. All gradients were linear.

**Appendix 7** Chromatograms for expanding and mature leaves for the six *Inga* species. The [M‒H]^‒^ ion of the internal standard, biochanin A at 22.5 min, has an *m/z* ratio of 283.0612 and, for nearly all runs, showed a mass error of less than 3.2 ppm. Species order is as in Table 1.

**Appendix 8** R code for statistical analyses of the LC-MS data. Analyses were performed using the provided programming language in the R Statistical Software Package. Code was directly adapted from the cited packages, with minor modifications applied.

**Appendix 9** Hierarchical cluster dendrograms of the metabolites of expanding and mature leaves from six species of *Inga* (*n*=5 samples per age class per species). The numbers above each branch-point are the Approximately Unbiased confidence levels; these indicate the probability that the samples below that point are a cluster. Clusters with values of 95 signify *P*=0.05, indicating that these are strongly supported by the data. Heights are 1x10^6^ for *I. marginata*, *I. auristellae, I. tenuistipula* and *I. laurina*, and 1x10^5^ for *I. acreana* and *I. umbellifera* from six species of *Inga*.

**Appendix 10** Two-dimensional scores scatter plot from a partial least squares-discriminant analysis fitted to the metabolite profiles of leaves sampled as a ‘paired sample collection’ versus a ‘non-paired sample collection’ (*n*=15 per group). Age class is represented by open (expanding) or closed (mature) symbols, and 95 % confidence intervals are indicated by dashed (expanding) or solid (mature) lines around each sample set. Values are derived from the total ion current of the features.

**Appendix 11** Insoluble metabolites assayed as condensed tannin equivalents. The quebracho standard was the crude form, meaning that it contains components other than condensed tannins, such as simple phenolics.

**Appendix 12** Toxicity of the marc for species of *Inga* from Barro Colorado Island, Panama.

**Appendix 1**

| Species | Sample ID | Leaf age class | Expansion (%) | Light conditions | Collection year |
| --- | --- | --- | --- | --- | --- |
| *Inga marginata* Willd. | e1 | expanding | 80 | shade | 2013 |
|  | e2 | expanding | 30-80 | shade | 2013 |
|  | e3 | expanding | 80 | shade | 2013 |
|  | e4 | expanding | 30 | shade | 2013 |
|  | e5 | expanding | 30-80 | intermediate | 2013 |
|  | m1 | mature | 100 | shade | 2013 |
|  | m2 | mature | 100 | intermediate | 2013 |
|  | m3 | mature | 100 | intermediate | 2013 |
|  | m4 | mature | 100 | shade | 2013 |
|  | m5 | mature | 100 | intermediate | 2013 |
| *Inga acreana* Harms | e1 | expanding | 50 | intermediate | 2013 |
|  | e2 | expanding | 20-30 | shade | 2013 |
|  | e3 | expanding | 40 | intermediate | 2013 |
|  | e4 | expanding | 20 | intermediate | 2013 |
|  | e5 | expanding | 50 | shade | 2014 |
|  | m1 | mature | 100 | shade | 2013 |
|  | m2 | mature | 100 | intermediate | 2013 |
|  | m3 | mature | 100 | intermediate | 2013 |
|  | m4 | mature | 100 | intermediate | 2013 |
|  | m5 | mature | 100 | shade | 2013 |
| *Inga auristellae* Harms | e1 | expanding | 50 | shade | 2013 |
|  | e2 | expanding | 40 | shade | 2013 |
|  | e3 | expanding | 80 | shade | 2013 |
|  | e4 | expanding | 30-60 | intermediate | 2014 |
|  | e5 | expanding | 50 | shade | 2014 |
|  | m1 | mature | 100 | shade | 2013 |
|  | m2 | mature | 100 | shade | 2013 |
|  | m3 | mature | 100 | sun | 2014 |
|  | m4 | mature | 100 | intermediate | 2014 |
|  | m5 | mature | 100 | shade | 2013 |
| *Inga tenuistipula* Ducke | e1 | expanding | 70 | intermediate | 2014 |
|  | e2 | expanding | 50 | intermediate | 2014 |
|  | e3 | expanding | 50 | shade | 2014 |
|  | e4 | expanding | 40-50 | shade | 2013 |
|  | e5 | expanding | 80 | shade | 2014 |
|  | m1 | mature | 100 | shade | 2013 |
|  | m2 | mature | 100 | shade | 2013 |
|  | m3 | mature | 100 | intermediate | 2014 |
|  | m4 | mature | 100 | intermediate | 2014 |
|  | m5 | mature | 100 | sun | 2013 |
| *Inga umbellifera* (Vahl) Steud ex DC. | e1 | expanding | 30-80 | sun | 2013 |
|  | e2 | expanding | 20-80 | shade | 2013 |
|  | e3 | expanding | 15 | sun | 2013 |
|  | e4 | expanding | 40 | shade | 2013 |
|  | e5 | expanding | 80 | intermediate | 2013 |
|  | m1 | mature | 100 | shade | 2013 |
|  | m2 | mature | 100 | intermediate | 2013 |
|  | m3 | mature | 100 | shade | 2013 |
|  | m4 | mature | 100 | shade | 2013 |
|  | m5 | mature | 100 | shade | 2014 |
| *Inga laurina* (Sw.) Willd. | e1 | expanding | 40 | intermediate | 2013 |
|  | e2 | expanding | 80 | shade | 2013 |
|  | e3 | expanding | 75 | shade | 2013 |
|  | e4 | expanding | 15-90 | sun | 2013 |
|  | e5 | expanding | 80 | intermediate | 2013 |
|  | m1 | mature | 100 | intermediate | 2013 |
|  | m2 | mature | 100 | shade | 2013 |
|  | m3 | mature | 100 | intermediate | 2013 |
|  | m4 | mature | 100 | intermediate | 2013 |
|  | m5 | mature | 100 | shade | 2014 |

**Appendix 2**

**

**

**Appendix 3**

| **Species** | **Leaf development stage** | **Median distance (m)**^1^  (Confidence Interval) |
| --- | --- | --- |
| *I. marginata* | All  Expanding  Mature | 991 (825 -1250)  946 (782 - 1580)  661 (318 - 1250) |
| *I. acreana* | All  Expanding  Mature | 1159 (998 - 1354)  1505 (998 - 1354)  1014 (398 - 1979) |
| *I. auristellae* | All  Expanding  Mature | 1043 (984 - 1213)  1013 (198 - 1275)  920 (828 -1457) |
| *I. tenuistipula* | All  Expanding  Mature | 1242 ( 1014 - 1511)  1131 (709 - 1536)  964 (596 - 1511) |
| *I. umbellifera* | All  Expanding  Mature | 1373 (1017 - 1457)  1746 (1133 -2645)  920 (828 - 1457) |
| *I. laurina* | All  Expanding  Mature | 887 (727 - 1116)  1131 (709 - 1536)  1002 (727 - 1255) |
| **All species** |  | **1087 (1049 - 1113)** |

^1^An effort was made to sample across 9 km^2^ in order to minimize spatial correlation. We report the median distance and the 95 % confidence interval.

**Appendix 4**

The chlorophyll content of leaves between 30 to 80% of full expansion was estimated using three values from a Minolta SPAD 502DL meter (Spectrum Technologies, Plainfield, IL, USA). For calibrating the SPAD, a field-portable Spectronic Mini 20 (Milton Roy, NY, USA) was first calibrated in the laboratory using expanding leaves. In the laboratory, leaves were extracted with 90% acetone/10% water (v/v) containing Na_2_CO_3_, and centrifuged at 10,000 x g at 5°C. Absorbances were obtained using a narrow bandpass spectrometer at 647 and 664 nm. Chlorophyll content was determined using the equations of Jeffrey & Humphrey (1975). For the laboratory calibration of the Spectronic Mini 20, the same leaves were extracted in 95% ethanol containing NaHCO_3_, Na_2_CO_3_, or Na_2_HPO_4_, centrifuged at 25°C in a mini-centrifuge (SC1006-R, Roebling, NJ), at 2,000 x g, and transmittance measured at 663 nm and 725 nm. Regression analysis gave the following equation for total chlorophyll *a* and *b* in units of mg m^‒2^ for the portable Spectronic Mini 20:

(1) chlorophyll = 152.4 * (A_663_-A_725_) (volume in mL)

(area in units of mm^2^)

where A_663_ and A_725_ are the absorbance readings for 663 nm and 725 nm, respectively. The SPAD meter was calibrated in the field using chlorophyll data from expanding leaves on the Spectronic Mini 20. Our SPAD calibration covers the range of 50 to 200 mg chl m^‒2^. The relationship is non-linear:

(2) total chlorophyll *a* and *b* in units of mg m**^‒2^** = a × SPAD**^b^**

where SPAD is the unitless reading from the SPAD, and a (0.0417) and b (0.9524) are the fitted parameters.

**Jeffrey SW, Humphrey GF. 1975.** New spectrophotometric equations for determining chlorophyll *a, b, c_1_* and *c_2_* in higher plants, algae, and natural phytoplankton. *Biochemie und Physiologie der Pflanzen* **167**: 191-194.

**Appendix 5**

**

**

**Appendix 6**

| **Time (min)** | **Flow rate (mL/min)** | **Mobile phase A (H_2_0 + 0.1 % FA^2^)^1^ %** | **Mobile phase B (ACN^3^ + 0.1 % FA)^1^ %** |
| --- | --- | --- | --- |
| 1.0  2.0  4.0  22.0  32.0  35.0  36.0  40.0  43.0  45.0 | 0.5  0.5  0.5  0.5  0.5  0.5  0.5  0.4  0.4  0.5 | 98  98  96  60  30  2  2  98  98  98 | 2  2  4  40  70  98  98  2  2  2 |

^1^All gradients were linear.

^2^Formic acid.

^3^Acetonitrile.

**Appendix 7**

**
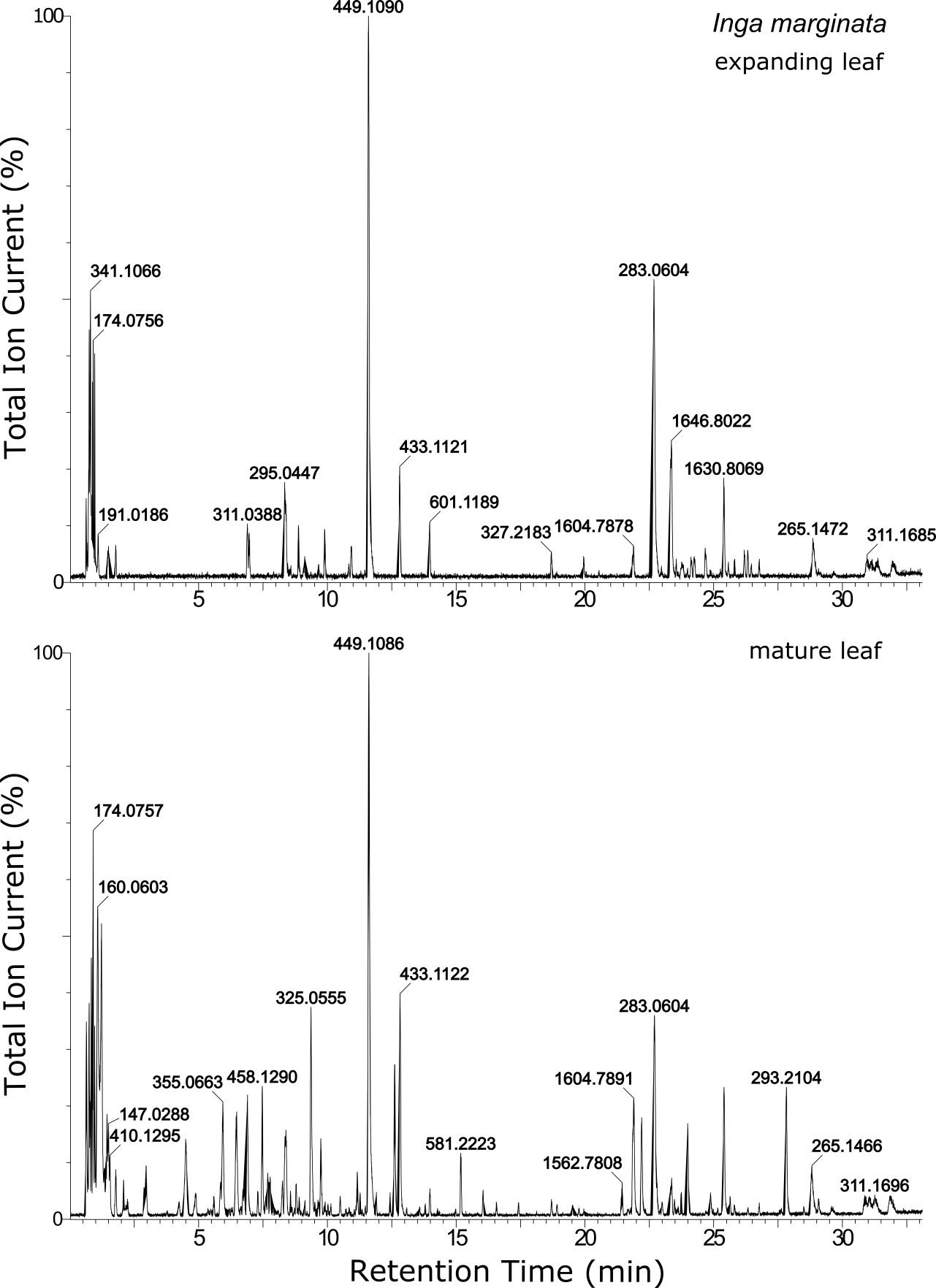
**

**
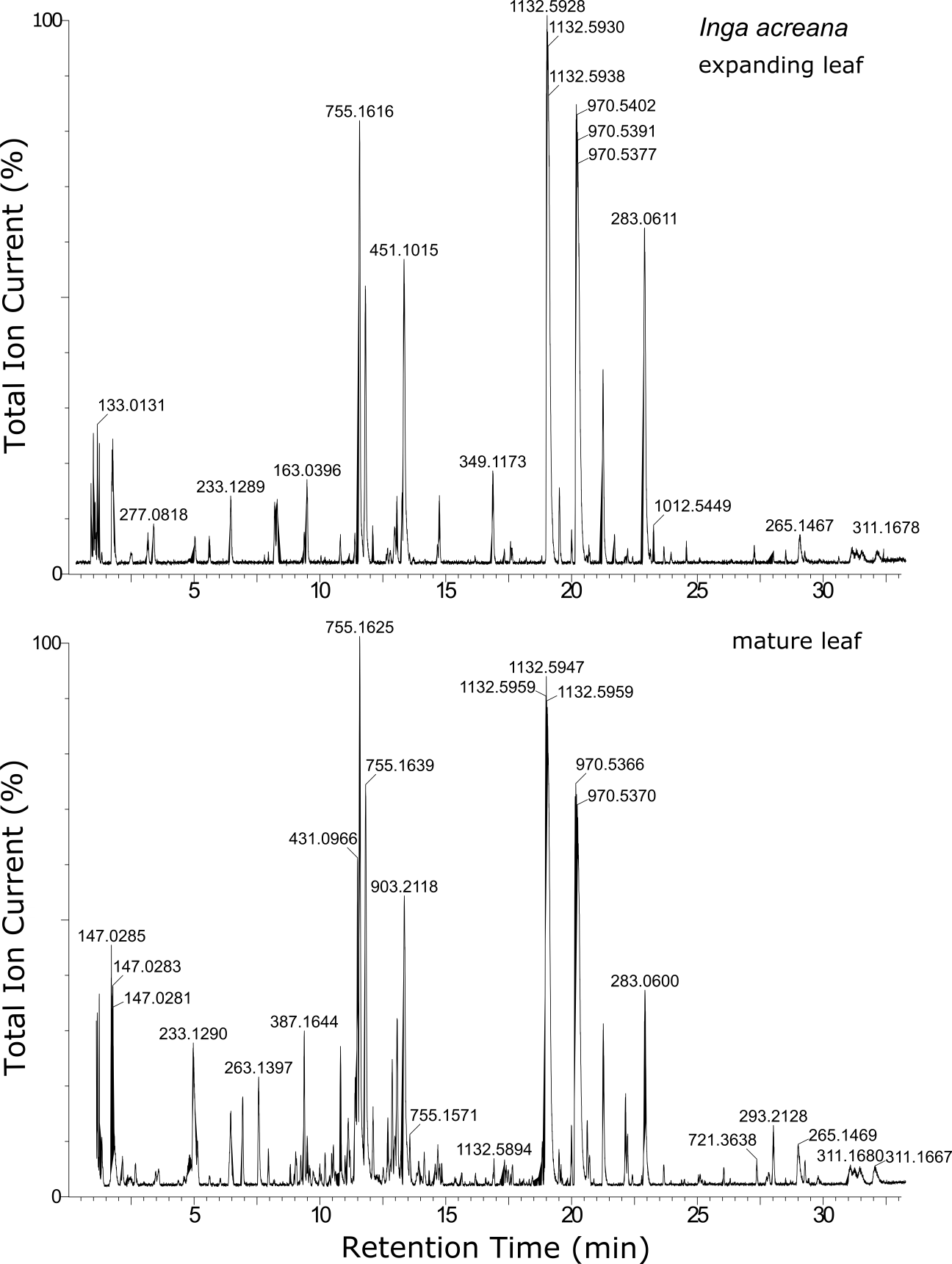
**

**
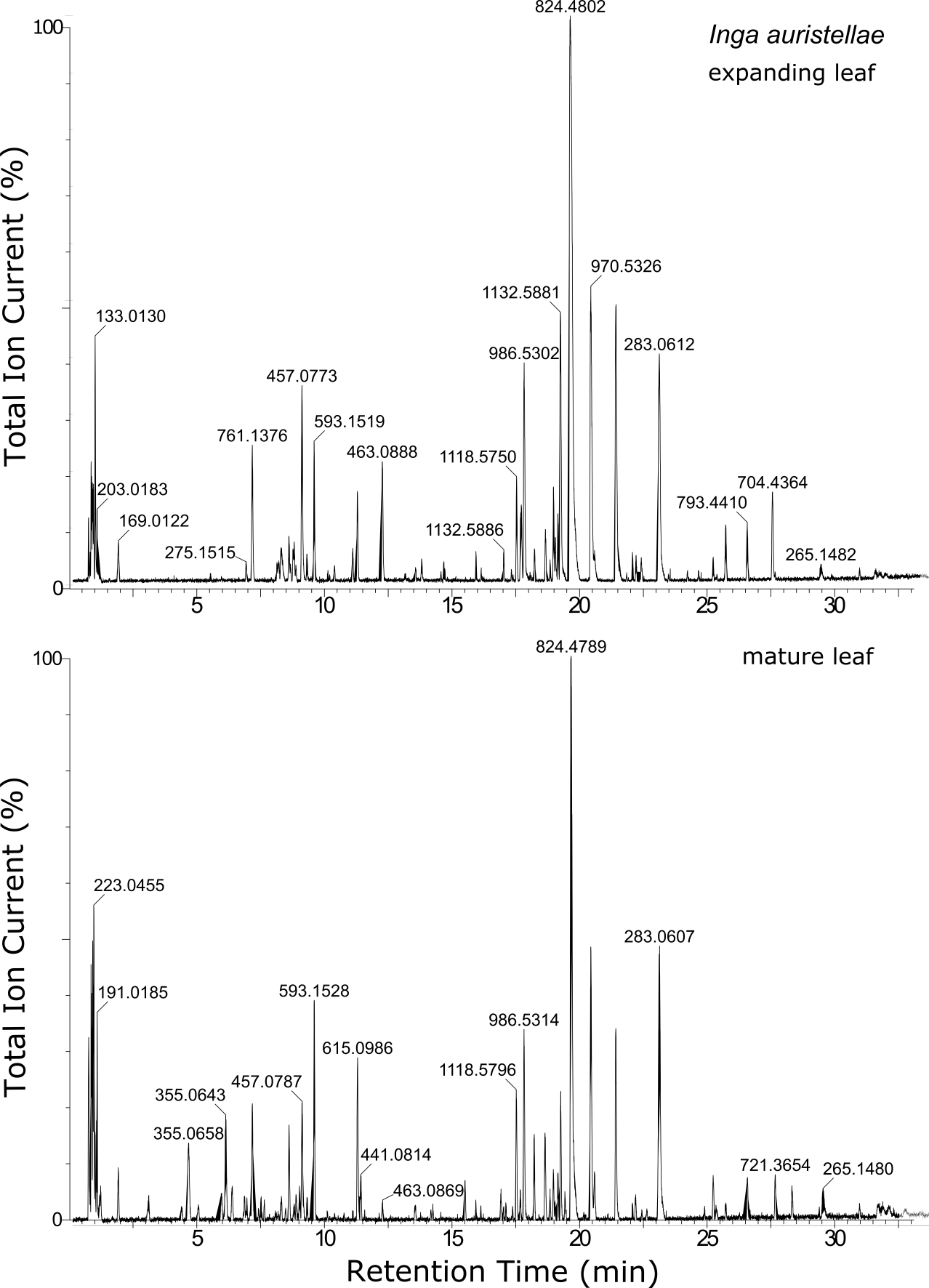
**

**
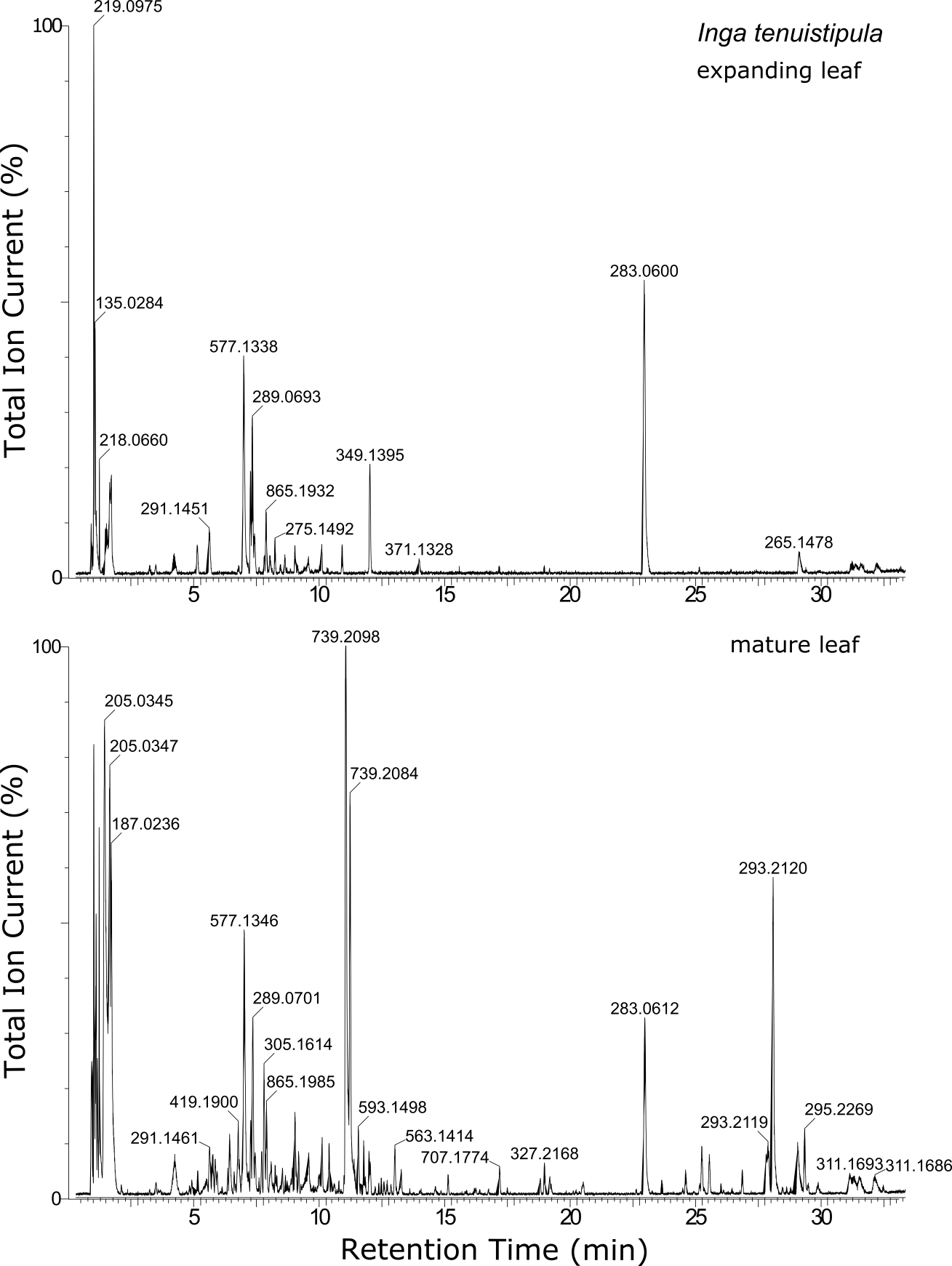
**

**
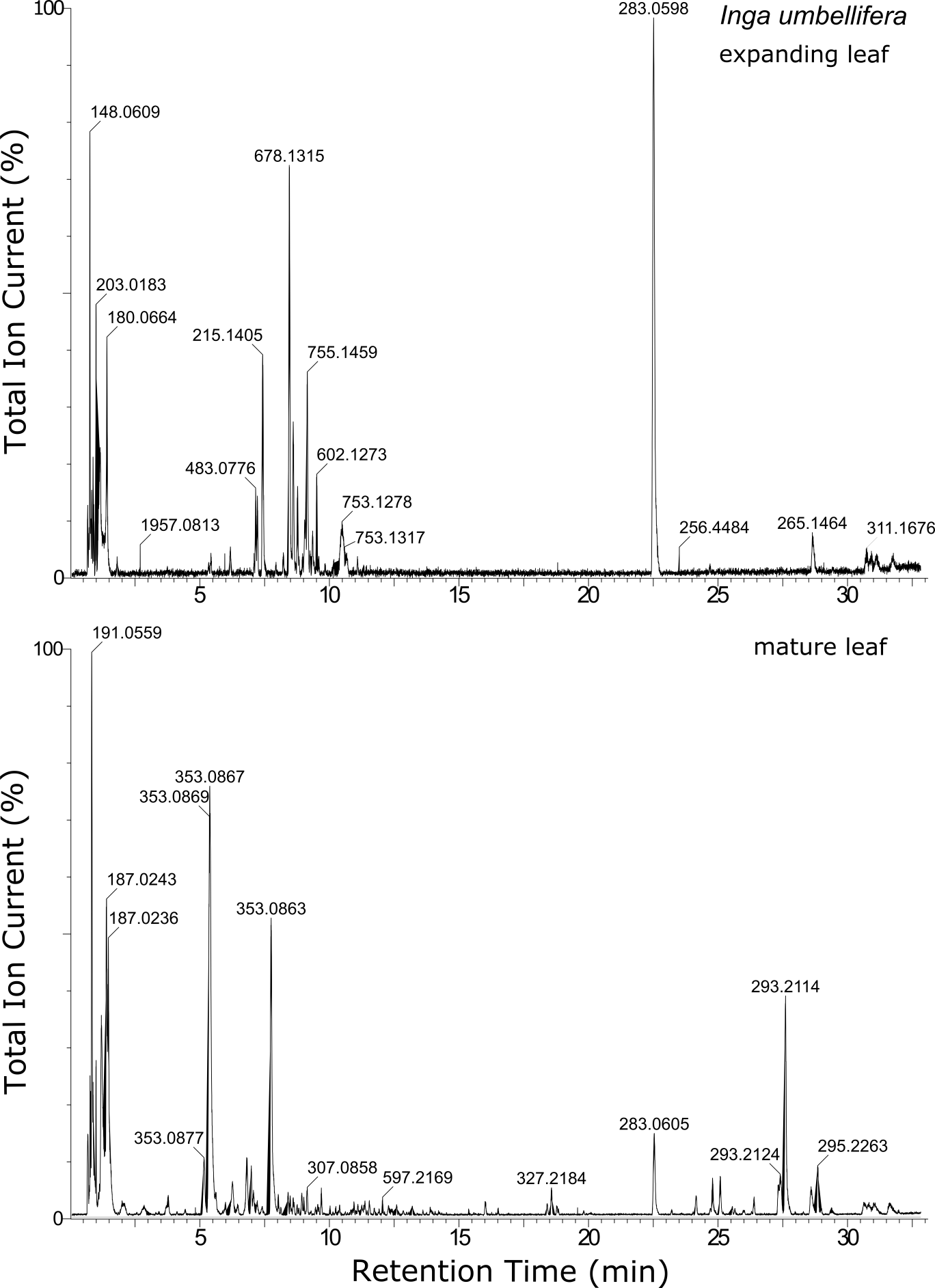
**

**
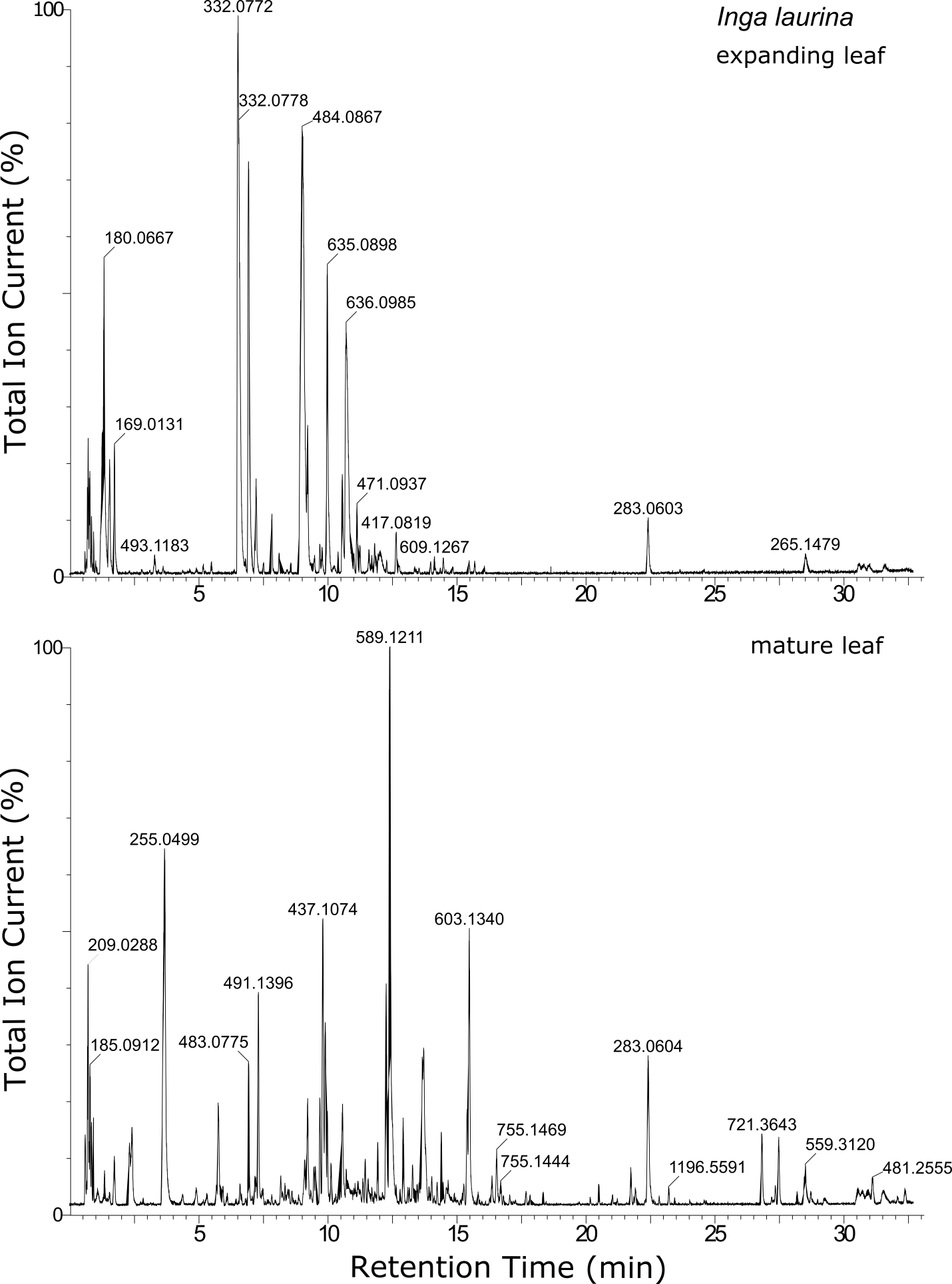
**

**Appendix 8**

------------------------------------------------------------

Data pre-processing: XCMS code for LC/MS raw data

------------------------------------------------------------

source("http://bioconductor.org/biocLite.R")

biocLite("xcms")

biocLite("multtest")

library(multtest) # Library preload

library(xcms)

xset <- xcmsSet(method='centWave',ppm=10,peakwidth=c(5,20), snthresh=4) # Peak identification

xset1 <-group(xset, method="density",bw=10, mzwid=0.05, minfrac=0.1) # Peak matching 1

xset2 <-retcor(xset1) # Retention time correction

xset3 <-group(xset2, method="density",bw=5, mzwid=0.05, minfrac=0.1) # Peak matching 2

xset4 <-fillPeaks(xset3) # Peak fill in

# Create data matrix with features without group assignment and p-values

peakTable <- function(xs){

if (nrow(xs@groups) > 0) {

groupmat <- groups(xs)

ts <- data.frame(cbind(groupmat,groupval(xs, "medret",

"into")),row.names = NULL)

cnames <- colnames(ts)

colnames(ts) <- cnames

} else if (length(xs@sampnames) == 1)

ts <- xs@peaks

else stop ('First argument must be a xcmsSet with group information

or contain only one sample.')

ts

}

xset5 <- peakTable(xset4) # Apply peakTable

xset5$rt_in_min<- (xset5$rtmed)/60 # Calculate retention time (min) write.table(xset5,file="peaktable2.csv",row.names=F, sep=",") # Visualizing data

----------------------------------------

Principal Component Analysis (PCA)

----------------------------------------

library(muma) # Library preload

data.filepath <- "E:/species_TIC" # Assign data filepath

species_TIC <- read.csv("species_TIC.csv", sep=",", header=TRUE) # Input data

explore.data (file="species_TIC.csv", scaling="Auto", scal=TRUE, normalize=TRUE, imputation=FALSE, imput="mean") # PCA

Plot.pca (1, 2, scaling="Auto", test.outlier = TRUE) # Plot first two components PCA

Plot.pca.score (1,2, scaling = "Auto") # Plot scores for the PCA components

-----------------------------------------------------------------------------------

Hierarchical Clustering with P-Values via Multiscale Bootstrap Resampling

-----------------------------------------------------------------------------------

library(pvclust) # Library preload

species_TIC <- read.csv("species_TIC.csv", header=TRUE) # Input data

species_dist <- as.dist(1-cor(species_TIC, method="pearson")) # Distance matrix

species_single <- hclust(species_dist, method="single") # Single linkage clustering

species_ward <- hclust(species_dist, method="ward.D") # Ward clustering

species_complete <- hclust(species_dist, method="complete") # Complete linkage clustering

species_centroid <- hclust(species_dist, method="centroid") # Centroid clustering

species_median <- hclust(species_dist, method="median") # Median clustering

#Comparison between the distance matrix and binary matrices representing partitions

coph1 <- cophenetic(species_single) # Compute Patristic distances

coph2 <- cophenetic(species_ward)

coph3 <- cophenetic(species_complete)

coph4 <- cophenetic(species_centroid)

coph5 <- cophenetic(species_median)

cor(coph1, species_dist) #Cophenetic correlations

cor(coph2, species_dist)

cor(coph3, species_dist)

cor(coph4, species_dist)

cor(coph5, species_dist)

# 'pvclust': Hierarchical cluster dendrogram with bootstrapping

result_species <- pvclust(species_TIC, method.hclust="ward", method.dist="euclidean", use.cor="pairwise.complete.obs", nboot=10000)

plot(result_species)

print(result_species)

**Appendix 9**

**
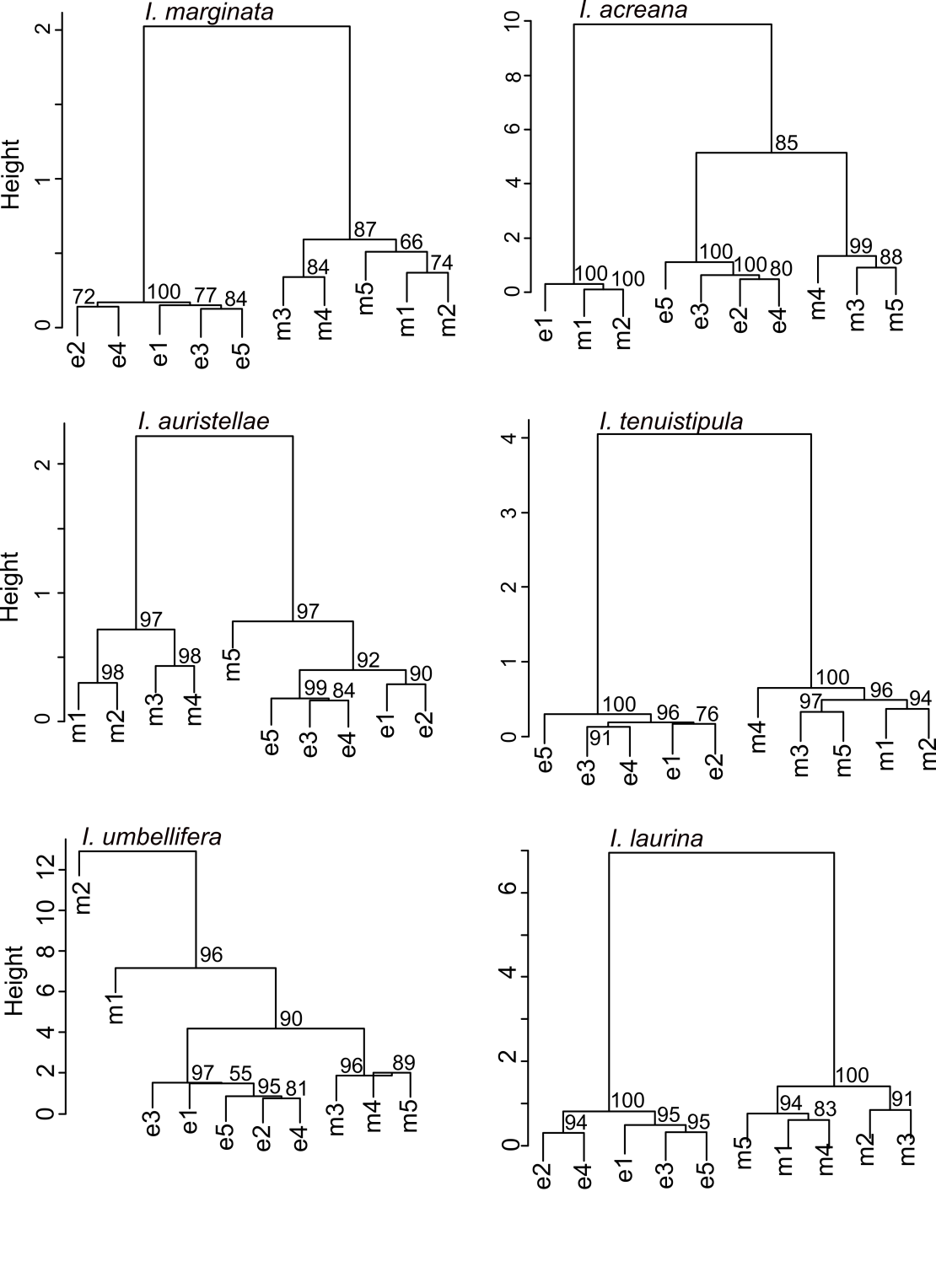
**

**Appendix 10**

**
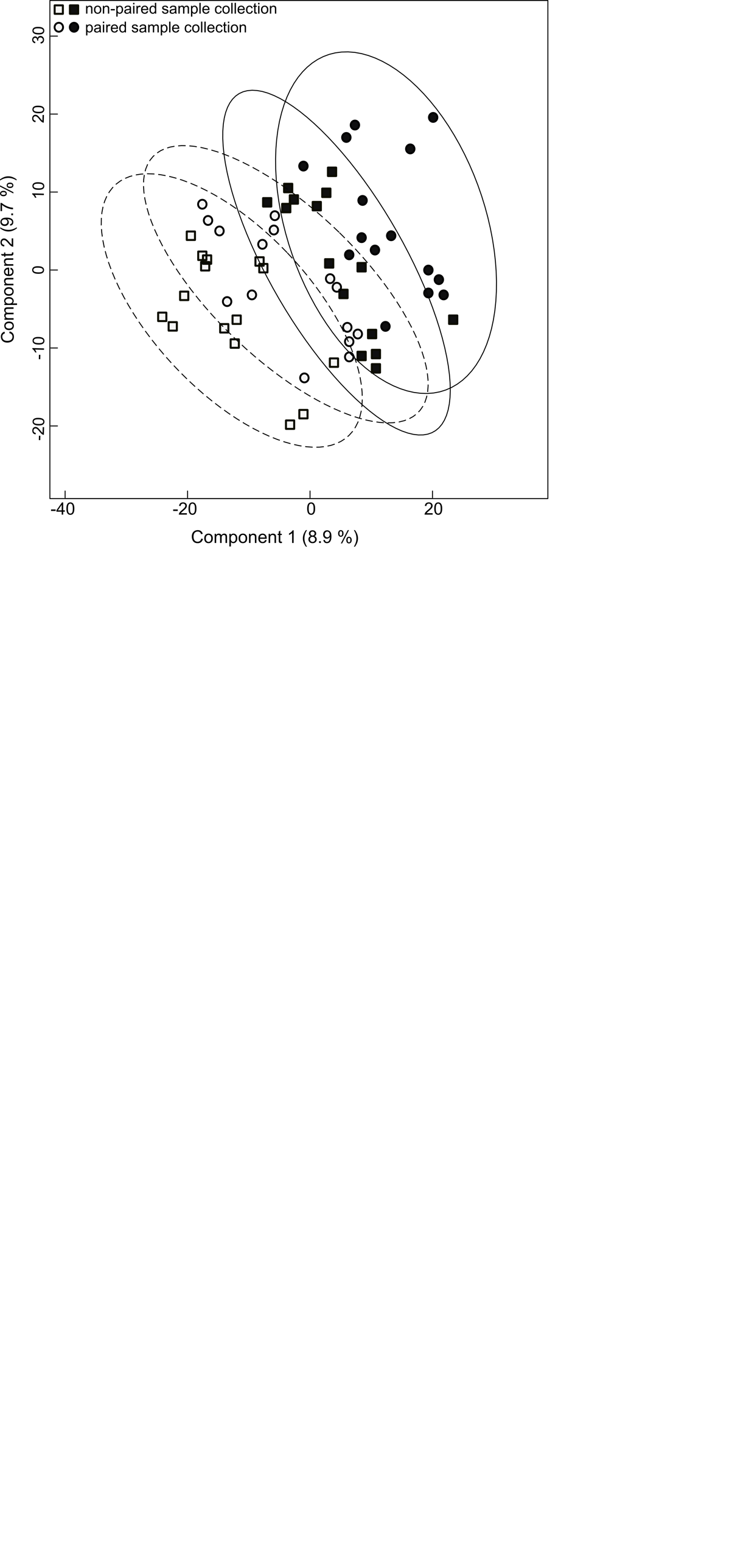
**

**Appendix 11**

| **Insoluble metabolites (% DW ± SE)** | | |
| --- | --- | --- |
| All Species | E | 3.45±0.70 |
|  | M | 3.48±0.54 |
| *I. marginata* | E | 0.35±0.29 |
|  | M | 3.17±0.14 |
| *I. acreana* | E | 5.77±1.18 |
|  | M | 5.78±0.59 |
| *I. auristellae* | E | 1.71±0.20 |
|  | M | 3.16±0.58 |
| *I. tenuistipula* | E | 5.81±0.96 |
|  | M | 3.93±0.41 |
| *I. umbellifera* | E | 3.63±0.32 |
|  | M | 3.00±0.36 |
| *I. laurina* | E | 0.30±0.12 |
|  | M | 1.82±0.16 |

**Appendix 12**

| **Species** | **Growth relative to control on crude marc** | **Data source** |
| --- | --- | --- |
| *I. acuminata* | 0.03 | *Kursar and Coley, unpublished* |
| *I. cocleensis* | 0.43 | *Kursar and Coley, unpublished* |
| *I. goldmanii* | 0.12 | Lokvam and Kursar (2005) |
| *I. laurina* | 0.75 | *Kursar and Coley, unpublished* |
| *I. marginata* | 0.16 | *Kursar and Coley, unpublished* |
| *I. thibaudiana* | 0.17 | *Kursar and Coley, unpublished* |
| *I. nobilis* | 0.22 | *Kursar and Coley, unpublished* |
| *I. pezizifera* | 0.14 | *Kursar and Coley, unpublished* |
| *I. sapindoides* | 0.84 | *Kursar and Coley, unpublished* |
| *I. umbellifera* | 0.12 | Lokvam and Kursar (2005) |
| *I. vera* | 0.25 | *Kursar and Coley, unpublished* |
| AVERAGE | 0.29 |  |
|  |  |  |
| **Species** | **Growth relative to control on extracted marc** | **Data source** |
| *I. goldmanii* | 0.69 | Lokvam and Kursar (2005) |
| *I. umbellifera* | 0.87 | Lokvam and Kursar (2005) |
| AVERAGE | 0.78 |  |

^1^Toxicity was quantified as ‘growth relative to control’ of lab-reared caterpillars *Heliothis virescens* (Lepidoptera: Noctuidae) on artificial diets. Growth was compared for 35 % of dry weight as marc relative to growth with 35 % of dry weight as cellulose (control diet). The ‘crude marc’ includes cell walls and insoluble metabolites and equals 35 % of dry weight in expanding leaves. Values range from 1.0, indicating growth similar to control, to substantially less than 1.0, indicating slower growth on crude marc than on control diet. Bioassays with crude marc extracted with butanol-HCl to remove the insoluble fraction (‘extracted marc’) showed a higher growth rate (less toxicity) than for the crude marc.

**Lokvam J, Kursar TA. 2005.** Divergence in structure and function of young leaf chemical defenses in two co-occurring *Inga* species. *Journal of Chemical Ecology* **31**: 2563-2580.
